# The needs and expectations of people eligible for lung cancer screening in Switzerland: A mixed method study

**DOI:** 10.1016/j.pmedr.2026.103382

**Published:** 2026-01-09

**Authors:** Mélinée Schindler, Cynthia Schneider, Christophe von Garnier, Cédric Bongard, Jean-Luc Bulliard, Chiara Pozzessere, Kevin Selby, Christina Akre

**Affiliations:** a University Center for Primary Care and Public Health (Unisanté), Department of Epidemiology and Health Systems, Route de la Corniche 10, 1010 Lausanne, Switzerland; b University Center for Primary Care and Public Health (Unisanté), Department of Outpatient Clinics, Route de la Corniche 10, 1010 Lausanne, Switzerland; c Division of Pulmonology, Department of Medicine, Lausanne University Hospital (CHUV) and University of Lausanne, Rue du Bugnon 46, 1011 Lausanne, Switzerland; d Department of Radiology, Lausanne University Hospital and University of Lausanne (CHUV), Rue du Bugnon 46, 1011 Lausanne, Switzerland

**Keywords:** Lung cancer, Screening, Risk communication, Switzerland, Implementation

## Abstract

**Objective:**

Organized lung cancer screening is being considered in Switzerland. This study assesses knowledge, barriers, and expectations regarding screening among the population and healthcare professionals prior to a pilot project.

**Methods:**

A convergent, mixed method approach combined an online survey, focus groups and interviews with laypeople aged 50–79 years with a smoking history and healthcare professionals. Data were collected between June and August 2023 in the Canton of Vaud, Switzerland.

**Results:**

Among 952 survey respondents (79% women, 60% current or ex-smokers), 19% were aware of lung cancer screening, but 75% would participate if offered.Responders preferred information from general practitioners (72%) or official letters (46%). In qualitative interviews, the 24 participants emphasized clear, non-stigmatizing, emotionally engaging communication, accessible logistics, and supportive professional guidance. Eligibility criteria were often viewed as too restrictive.

**Conclusions:**

The implementation of lung cancer screening in Switzerland will require a multichannel communication campaign, training and tools for healthcare professionals, and clear eligibility criteria using a pedagogical approach.

## Highlights

• Study of lung cancer screening implementation in Switzerland.
• Awareness of lung cancer screening was low, but willingness to participate was high.
• Participants call for clear, non-stigmatizing information via primary care doctors.
• Implementation requires multichannel communication and smoking cessation support.

## 1 Introduction

Lung cancer remains the leading cause of cancer mortality worldwide, with 1.8 million deaths in 2022 (Bray et al., 2024) and a projected 20% increase by 2040 (European Cancer Information System. Long term estimates of cancer incidence and mortality, for all countries [Internet], n.d.). In Switzerland, it is the leading cause of cancer deaths in both sexes (3600 annual deaths) (Federal Statistical Office, 2021), most diagnosed at an advanced stage. Smoking prevalence has decreased among men, but remains high overall, especially in socioeconomically disadvantaged groups, sustaining health inequalities (Federal Statistical Office. Consommation de tabac, envie d'arrêter de fumer, tabagisme passif (Tobacco use, desire to quit smoking, secondhand smoke). [Internet], n.d.).

Low-dose computed tomography (LDCT) screening can reduce lung cancer mortality by 20–25% among high-risk smokers (Bonney et al., 2022). However, concerns about overdiagnosis, false positives, and radiation exposure highlight the need for structured programs with clear guidelines (Bonney et al., 2022; Pozzessere et al., 2023). In 2022, the European Union and the Swiss Cancer Screening Committee recommended organized lung cancer screening (Cancer Screening Committee. Lung Cancer Screening using LDCT [Internet], n.d.). The “Cancer Care Monitor 2022” survey also revealed public support for strengthening lung cancer prevention and early detection (GFS.Bern, 2022).

The success of lung cancer screening in Switzerland will depend on effectively reaching the eligible population. Lung cancer screening participation rates vary widely (Crosbie et al., 2019; Jungblut et al., 2023). Facilitators include proactive health behavior, a positive perception of the accuracy of testing (Maki et al., 2021; Patel et al., 2012) and organized programs, including personalized invitations (Crosbie et al., 2019). Obstacles such as costs not covered by insurance, location of examinations, and waiting times remain significant barriers (Balata et al., 2019; Zhao et al., 2021; Broekhuizen et al., 2017). Referrals from trusted healthcare professionals are essential to motivate the target population (Kee et al., 2021; Rollet et al., 2021; Byrne et al., 2019). Digital campaigns may also improve engagement (Jessup et al., 2018).

Numerous lung cancer screening pilot projects are underway in Europe, while the United Kingdom is scaling up and Croatia already has a national program (Adams et al., 2023). In 2023, the Canton of Vaud launched the first population-based pilot in Switzerland. In preparation for this initiative and, eventually a future structured program, this preparatory study aimed to inform the pilot's design and communication strategies by assessing: (Bray et al., 2024) participants' knowledge and awareness of early detection methods for lung cancer; (European Cancer Information System. Long term estimates of cancer incidence and mortality, for all countries [Internet], n.d.) factors that make people reluctant or unmotivated to undergo screening; and (Federal Statistical Office, 2021) public expectations in terms of communication, type of information and support they would consider useful for an organized screening program.

## 2 Methods

A convergent mixed method approach was chosen, to qualitatively explore attitudes and perceptions in depth, while quantifying their prevalence within the target population. This research has been authorized by the Vaud Cantonal Human Research Ethics Committee (BASEC: 2023–00849). Data were collected between June and August 2023 in the Canton of Vaud, Switzerland.

### 2.1 Population

#### 2.1.1 Sampling

To assess participants' knowledge of lung cancer early detection method, an anonymous online survey was distributed via Facebook and Instagram, both selected for their broad reach and demographic targeting capabilities. A targeting algorithm was used to reach users aged 50 and over who were likely to be current or former smokers, based on profile data (e.g., age, declared interests) and online behavior (e.g., interactions with content related to smoking, cessation, or tobacco-related diseases). Geographic targeting focused on Lausanne and surrounding areas, where the lung cancer screening pilot project in the canton of Vaud was planned. We included all responses from residents of Canton Vaud aged older than 18 years, regardless of their smoking status. Sex was self-reported and used in descriptive analyses; gender was not assessed.

The qualitative component included both potential screening participants and healthcare professionals, with a balanced sex distribution. Participants from the general population were recruited among survey respondents aged 50 to 79 years who were current or former smokers and agreed to be recontacted. Healthcare professionals were selected based on predefined criteria, including their occupation (physician, nurse, or pharmacist) and their practice setting (urban or rural). They were recruited through networks or prior involvement in screening.

### 2.2 Quantitative study

The online survey was administered via REDCap®. It comprised 26 questions and took approximately 10 min to complete. The survey included: sociodemographic characteristics (questions 1 to 6), tobacco exposure and eligibility for screening (*Federal Statistical Office. Enquête suisse sur la santé*, 2022) (), and knowledge of lung cancer screening and information sources (). It also assessed previous experiences with lung cancer or other screening programs (). Questions 15 to 24 focused on willingness to undergo screening and underlying motivations, while question 25 addressed acceptability of a smoking cessation consultation. Finally, question 26 measured participants' willingness to take part in follow-up interviews. Several questions were adapted from previously published surveys to enhance comparability and reproducibility (Selby et al., 2020; See et al., 2020; Diab Garcia et al., 2022). The survey was pilot-tested with five people between the ages of 50 and 79 years. All quantitative analyses were conducted using Stata® version 17 (StataCorp LLC, College Station, TX, USA). Completion of the anonymous survey constituted informed consent.

### 2.3 Qualitative study

To identify factors that influenced the decision to undergo screening and to test communication tools, we conducted a qualitative study interviewing people potentially eligible for lung cancer screening and health professionals. After giving their agreement in principle, participants were contacted again to be informed in more detail about the qualitative part of the study and to receive the consent form.

Interviews were guided by the COM-B model (Capability, Opportunity, Motivation, Behavior) (Michie et al., 2011; Atkins et al., 2017), a framework for participants' perceptions, attitudes, and behaviors regarding lung cancer screening. Three interview guides were created, all including the following main themes: factors influencing the decision to undergo lung cancer screening, information needs, and the links between screening and smoking cessation. The three guides differed in the scenarios and the description of the health professionals' activities.

All interviews were recorded and transcribed verbatim. Based on the transcripts, we conducted a deductive thematic analysis (Konstantinos, 2022) using a codebook. The latter was created by systematically coding all the data and categorizing the codes based on the elements of the interview grid. A total of 10% of all data were double coded. We then themed the categories based on perception of communication tools, motivations and barriers to screening and role in prevention.

### 2.4 Integration of results

A convergent mixed method approach was adopted to cross-analyze quantitative and qualitative data (Ruffin et al., 2009; *Designing and Conducting Mixed Methods Research. By John W. Creswell and Vicki L. Plano Clark. Thousand Oaks, CA: Sage Publications, 2007. ISBN: 1–4129–2791-9. Library, and Information Science Research,* 2007; Pluye and Hong, 2014). The quantitative and qualitative data were first analyzed separately. Integration was conducted after separate analyses through an iterative comparison process, whereby qualitative themes and quantitative findings were repeatedly examined in relation to each other to identify patterns of alignment, contradiction, and complementarity.

## 3 Results

### 3.1 Quantitative results

#### 3.1.1 Respondent profiles

A total of 952 individuals completed the survey, the majority of whom were women (79%) and current or former smokers (60%), primarily aged between 50 and 69. Most participants had a secondary or higher level of education. Current and former smokers were slightly more often men (22%) than non-smokers (20%) and tended to have a lower level of education than non-smokers, with a higher representation of participants reporting an apprenticeship as their highest qualification (35% vs 19%). Demographic characteristics of the respondents are reported in Table 1.

**Table 1:** Descriptive sociodemographic characteristics of adults aged 50–79 years who completed the online survey on lung cancer screening in the Canton of Vaud, Switzerland, June–August 2023.

	All *n (column %)*	Non-smokers *n (row %)*	Smokers or ex-smokers *n (row %)*
*N (%)*	952 (100)	381 (100.0)	571 (100.0)
Age (years)			
18–39	94 (9.9)	50 (13.1)	44 (7.7)
40–49	84 (8.8)	40 (10.5)	44 (7.7)
50–59	452 (47.5)	188 (49.3)	264 (46.2)
60–69	249 (26.2)	77 (20.2)	172 (30.1)
70–79	64 (6.7)	21 (5.5)	43 (7.5)
> 80	9 (0.9)	5 (1.3)	4 (0.7)
Gender			
Male	203 (21.3)	75 (19.7)	128 (22.4)
Female	748 (78.6)	306 (80.3)	442 (77.4)
Other	1 (0.1)	0 (0.0)	1 (0.2)
Place of residence			
Large city (>100,000 inhabitants)	279 (29.3)	96 (25.2)	183 (32.0)
Medium-sized city (10,000 to 100,000 inhabitants)	262 (27.5)	108 (28.3)	154 (27.0)
Village (<10,000 inhabitants)	342 (35.9)	149 (39.1)	193 (33.8)
Rural area	69 (7.2)	28 (7.3)	41 (7.2)
Level of education attained			
Compulsory schooling or less	31 (3.3)	9 (2.4)	22 (3.9)
Apprenticeship	272 (28.6)	74 (19.4)	198 (34.7)
Upper secondary school diploma (Swiss Maturity)*	96 (10.1)	40 (10.5)	56 (9.8)
Higher education or university	551 (57.9)	256 (67.2)	295 (51.7)
Don't know	2 (0.2)	2 (0.5)	0 (0.0)
Country of origin			
Switzerland	710 (74.6)	274 (71.9)	436 (76.4)
France	75 (7.9)	21 (5.5)	54 (9.5)
Other countries	167 (17.5)	86 (22.6)	81 (14.2)
			

Note: n: number; %: percentage, * Maturity refers to the Swiss upper secondary school diploma granting access to university.

#### 3.1.2 Smoking behavior and cessation

Nearly two thirds of respondents reported having smoked at some point in their lives (34% former smokers, 26% current smokers), with an average tobacco exposure of 23 pack-years (median = 17). Among current smokers, 75% said they would be willing to receive cessation advice as part of the screening process. Among the 25% who declined, the main reasons given were: already receiving help, no desire to quit, or discomfort discussing smoking with a healthcare professional. Results are presented in Table 2.

**Table 2:** Distribution of smoking status and smoking cessation behaviors among adults aged 50–79 years who completed the online survey on lung cancer screening in the Canton of Vaud, Switzerland, June–August 2023.

	All	Non-smokers *n (%)*	Ex-smokers *n (%)*	Smokers *n (%)*	
	952	325 (34.1)	381 (40.0)	246 (25.8%)	
				Yes *n (%)*	No *n (%)*
Would you be willing to receive advice on quitting smoking as part of lung cancer screening?	235			176 (74.9)	59 (25.1)
					Selected *n (%)*
If not, is there a particular reason?	65				5 (7.7)
I already have the help I need to try and stop					27 (41.5)
I'm not at all interested in trying to quit right now.					4 (6.2)
I think it's a waste of time					14 (21.5)
I don't need help to stop smoking					6 (9.2)
I don't feel comfortable talking to a healthcare professional about my smoking habits.					9 (13.8)

Note: n: number; %: percentage.

#### 3.1.3 Knowledge of lung cancer screening

When asked whether participants were aware of a test to detect lung cancer at an early stage, a total of 67.4% of participants admitted they did not know, 13.4% answered incorrectly (for example, by mentioning chest X-rays, blood tests, or other non-recommended methods), and only 19.2% were aware such a test existed. Among those who were aware, 57% correctly identified LDCT scan as the preferred method for early detection.

#### 3.1.4 Preferred information sources

General practitioners were the most trusted source of information, rated by 72% of participants as “very useful,” followed by official letters from the cantonal health authorities (46%). Social media platforms received mixed reactions: about one-quarter of respondents considered them useful, while another quarter considered them not useful. Other channels such as pharmacists, television programs, insurance company mailings, or newspaper articles received more neutral evaluations. Results are available in Table 3.

**Table 3:** Descriptive distribution of perceived usefulness of different information sources among adults aged 50–79 years who completed the online survey on lung cancer screening in the Canton of Vaud, Switzerland, June–August 2023.

	All	Not at all useful *n (%)*	Not useful *n (%)*	Neutral *n (%)*	Useful *n (%)*	Very useful *n (%)*
Via social media (e.g. Facebook, Twitter, Instagram)	886	239 (27.0)	138 (15.6)	146 (16.5)	131 (14.8)	232 (26.2)
Talking to my primary care doctor	923	13 (1.4)	15 (1.6)	45 (4.9)	186 (20.2)	664 (71.9)
Talk to your pharmacist	876	130 (14.8)	155 (17.7)	220 (25.1)	178 (20.3)	193 (22.0)
By receiving a phone call from the township or from my general practitioner's office	882	127 (14.4)	102 (11.6)	177 (20.1)	191 (21.7)	285 (32.3)
When I received a letter from my health insurance company	887	183 (20.6)	129 (14.5)	168 (18.9)	175 (19.7)	232 (26.2)
On receiving a letter from the Canton of Vaud	900	61 (6.8)	50 (5.6)	136 (15.1)	237 (26.3)	416 (46.2)
Watching a local news or special program on TV or listening to the radio	884	98 (11.1)	125 (14.1)	238 (26.9)	238 (26.9)	185 (20.9)
Reading an article about it in a magazine/newspaper	884	66 (7.5)	126 (14.3)	246 (27.8)	249 (28.2)	197 (22.3)

Note: n: number; %: percentage.

#### 3.1.5 Attitudes toward screening

A large majority of respondents (75%) indicated they would undergo lung cancer screening if it were offered. The main motivation (64%) was the desire for reassurance regarding the absence of disease. Only 14% expressed interest in screening primarily to receive help with smoking cessation.

Potential barriers identified in the survey appear to be limited: around 8% were concerned about radiation exposure, and only 20% reported other health issues or time constraints as major obstacles. Notably, 20% reported they would feel disappointed if found ineligible, suggesting an emotional investment in having access to screening. Results are presented in Table 4.

**Table 4:** Descriptive distribution of intentions and perceived barriers regarding participation among adults aged 50–79 years who completed the online survey on lung cancer screening in the Canton of Vaud, Switzerland, June–August 2023.

	All	Strongly disagree *n (%)*	Disagree *n (%)*	Neither agree nor disagree *n (%)*	Agreed *n (%)*	Totally agree *n (%)*
I want screening to reassure me that I do NOT have lung cancer	857	104 (12.1)	65 (7.6)	137 (16.0)	132 (15.4)	419 (48.9)
I do NOT want screening because I have too many other medical problems.	774	631 (81.5)	66 (8.5)	47 (6.1)	15 (1.9)	15 (1.9)
I do NOT want to be screened because I am concerned about the radiation emitted by the scanner.	785	556 (70.8)	86 (11.0)	81 (10.3)	41 (5.2)	21 (2.7)
I do NOT want a screening because I would be too worried, anxious or stressed.	781	506 (64.8)	139 (17.8)	98 (12.5)	23 (2.9)	15 (1.9)
I do NOT want to be screened because it would be too difficult for me to travel to the Lausanne University Hospital.	778	672 (85.9)	43 (5.7)	35 (4.7)	11 (1.4)	17 (2.3)
I do NOT want a screening because it would be too difficult for me because of my work or other obligations.	776	626 (80.2)	68 (8.8)	48 (6.2)	22 (3.2)	12 (1.6)
I want to be screened to get help to stop smoking	749	453 (60.3)	50 (6.8)	90 (12.1)	49 (6.6)	107 (14.2)
I would be disappointed if someone told me I was NOT eligible.	781	309 (39.2)	68 (8.6)	148 (19.1)	99 (12.9)	157 (20.2)
I would do a lung cancer screening if it were offered to me	846	66 (7.8)	41 (4.8)	108 (12.6)	143 (16.9)	488 (57.9)

Note: n: number; %: percentage.

### 3.2 Qualitative results

#### 3.2.1 Respondent profiles

The qualitative study involved 24 participants from the canton of Vaud: 18 from the general population, eight in a focus group and 10 in individual interviews, and six individual interviews with healthcare professionals - two physicians, two nurses, two pharmacists. The participants varied in age (36–75 years), education level, and experience with other cancer screenings. These results are presented in Table 5.

**Table 5:** Descriptive sociodemographic characteristics of adults and healthcare professionals participating in focus groups and individual interviews on lung cancer screening in the Canton of Vaud, Switzerland, June–August 2023.

Participants	Focus group with members of the population	Interviews with members of the population	Interviews with healthcare professionals
*N* = 24	*n* = 8	*n* = 10	*n* = 6 2 doctors (general practitioner and pulmonologist) 2 nurses (primary care and cardiovascular prevention) 2 pharmacists (rural and urban)
Gender (women)	4	5	3
Age	between 48 and 75	between 36 and 58	between 52 and 75
Education level	1 = compulsory school 2 = professional training 5 = high school or university	1 = compulsory school 3 = professional training 6 = high school or university	6 = high school or university
Experience of breast, colon and prostate cancer screening / or of promotion among health professionals	6 = yes (4 women) 2 = no	7 = yes (4 women) 3 = no	6 = (promotion experience)
Experience of lung cancer screening / or of promotion among health professionals	0	1	1 (promotion experience) 3 (no promotion experience)

Note: n: number.

#### 3.2.2 Perception of communication tools

Participants from the general population emphasized the importance of receiving information on lung cancer screening that is clear, accessible, and non-stigmatizing, focusing on the benefits of early detection, such as avoiding systemic therapy and preserving quality of life: *“You really need to highlight that early detection reduces risk.”.* Emotion, especially through personal testimonials, was viewed as a powerful motivator: *“Testimonials from people who've been through it can really help.”*to identify with the people concerned and understand that it is possible to survive in good conditions after early detection.

The complexity of eligibility criteria was a source of confusion, and participants from general population called for simplification.Criteria were often seen as too restrictive or arbitrary: *“What's a heavy smoker anyway? These people should be tested too.”.* Screening only active or long-term smokers was perceived as unfair by some participants and professionals, particularly in the case of a positive family history.

General practitioners, pulmonologists, and official letters were seen as the most trustworthy sources. Social media was viewed as underused but potentially useful, if the tone avoids judgment: *“Being moralistic doesn't help… if someone doesn't want help, it won't work.” (healthcare professional).* The emphasis is on the repetition and visibility of prevention messages through multiple channels.

Healthcare professionals found the materials clear and relevant, but time constraints limited their use in practice. They also noted that lung cancer screening is not yet a routine topic in consultations and expressed a need for additional tools and training to better support communication.

#### 3.2.3 Motivations and barriers to screening

The primary motivation for screening in the general population was a perception of personal risk related to smoking, respiratory symptoms, chronic illness, or family history: *“I started coughing in a way that worried me… I told myself I had to act.”.* Smoking was a central concern too: *“Lung cancer is linked to smoking, people take that risk.”.* But other factors were also mentioned: *“It's not just smoking, there's the environment, work exposure too.”.* Personal experiences, such as the illness of a non-smoking relative, also influenced risk perception: *“My father died of lung cancer… he was athletic, never smoked.”.* Facilitators included recommendations from trusted healthcare providers, visible awareness campaigns, accessible screening sites, and free testing.

Barriers included fear of diagnosis, feelings of guilt or stigma, and administrative burden: *“Making appointments is hard… too much paperwork.”.* Cost was also cited as a disincentive: *“They don't pay for prevention, but they'll pay for treatment… it's absurd.”*, referring to the healthcare insurance, obligatory in Switzerland.

#### 3.2.4 Roles in prevention

All participants agreed on the central role of healthcare providers, especially general practitioners, in promoting screening: *“The closer the provider is to the patient, the easier it is to talk about it”*, *“We set goals together… I try to build an alliance with my patients.” (healthcare professionals).* While prevention was often seen as the responsibility of the healthcare system, some participants from the general population were open to a more active role when supported by a trusted professional: *“It's through dialogue with your doctor that you build that partnership.”.* Both groups highlighted the importance of collaboration and appropriate support to encourage engagement in prevention and screening.

### 3.3 Result integration

The mixed method approach allowed for the integration of general trends with lived experiences, providing a richer interpretation of the findings. Both components converged on the observation of limited awareness about lung cancer screening, yet strong acceptability once informed, as well as on the central role of general practitioners and the need for clear, non-judgmental, and multimodal communication.

However, qualitative data provided important nuances, particularly regarding the intention to undergo screening, concerns about the examination, and smoking cessation. While the intention to undergo screening was high, it appeared fragile when confronted with barriers such as fear of diagnosis, stigma related to smoking, or the perceived complexity of eligibility criteria.

Several paradoxes also emerged: although logistical issues or exposure to scanning were rarely identified as concerns in the survey, interviews revealed concrete anxieties related to the exam itself or the travel required. Similarly, although smoking cessation was not often cited as a motivation in the quantitative data, interviews suggested that screening could serve as an emotional trigger to initiate such a process.

The integration of both approaches thus shows that apparent acceptability may mask more subtle barriers, reinforcing the value of designing screening programs grounded in the lived experiences of the target population.

## 4 Discussion

This study shows that risk perception, information quality, and understanding of eligibility criteria shape adherence to lung cancer screening in Switzerland. The mixed method approach enabled the iterative comparison process and co-construction of results.

A key finding was the gap between low awareness of screening and high acceptability once informed. This result, consistent with other studies (Zhao et al., 2021; See et al., 2020), indicated that the main lever lies in clear and accessible information, more than in persuasive strategies. The lack of visibility of lung cancer screening compared to other cancer screening programs underlines the need for a structured, multimodal communication strategy.

Social representations of smoking produced ambivalent effects: while some feel concerned, others anticipate a judgmental message, which can deter them. Such mechanisms of stigma, previously documented (Maki et al., 2021; Rollet et al., 2021), argue in favor of empathetic and non-moralizing campaigns. No major limitations were seen. However, the survey highlighted the importance of informing participants about the risk-to-benefit balance of radiation exposure of LDCT in lung cancer screening.

The role of healthcare professionals was confirmed as a key driver in motivating participation (Kee et al., 2021; Byrne et al., 2019). However, their involvement was limited by time constraints and lack of resources. Tailored support was needed, along with complementary strategies such as official invitation letters or messages via health insurers (Walsh et al., 2011).

Qualitative findings also revealed a discrepancy between the public health logic of eligibility criteria and individual perceptions of risk. Although scientifically grounded (Bonney et al., 2022; de Koning et al., 2020), the criteria were sometimes perceived as unfair. This highlights the importance of pedagogical and transparent communication to preserve trust. Specific care should be taken to train healthcare professionals to deliver messages about the risks of screening and that these risks (i.e. radiation exposure, false-positives and incidental findings) likely outweigh the benefits for lower risk individuals. While there is ongoing research exploring the efficacy of screening based on other risk factors (Wu et al., 2016; Chang et al., 2024), especially in Asian populations, there is currently insufficient evidence to warrant exposing individuals without a heavy exposure to tobacco.

Data integration revealed discrepancies between intentions and realities. Interviews highlighted concrete barriers, such as anxiety and logistics, not visible in the quantitative data. This kind of dissonance, frequently observed in mixed method research (Ruffin et al., 2009; Pluye and Hong, 2014), underscores the need for interventions rooted in real-life experiences.

Based on the integrated findings, several key recommendations for the implementation of lung cancer screening were identified in Table 6.

**Table 6:** Implementation recommendations for lung cancer screening derived from survey and interview data in adults and healthcare professionals in the canton of Vaud, Switzerland (2023–2024).

Recommendation	Description
Multichannel, non-stigmatizing communication	Develop communication campaigns that highlight screening benefits using clear, supportive, and non-judgmental messages.
Support for healthcare professionals	Provide training and practical tools to healthcare professionals, particularly general practitioners, to facilitate patient dialogue.
Clear eligibility criteria	Clarify eligibility criteria to reduce misunderstanding and feelings of exclusion among potential participants.
Facilitated access to screening	Improve access to screening services for geographically or socioeconomically disadvantaged groups.
Optional emotional and cessation support	Offer optional emotional support and smoking cessation assistance as part of the screening pathway.

Note: These recommendations directly informed the design of the Vaud pilot lung cancer screening program.

### 4.1 Strengths and limitations

The main strength is in its mixed method design, reinforcing the validity of recommendations. The large number of respondents and the diversity of participant profiles are also notable strengths.

However, recruiting via social media likely introduced selection bias, potentially excluding less digitally connected populations. The strong female predominance among survey respondents (79%) may limit generalizability, reflecting gender differences in online surveys participation (de Koning et al., 2020) and preventive health engagement, which could amplify positive attitudes toward screening. Future studies should diversify recruitment to achieve better gender balance. The online distribution also prevented calculation of a response rate and may have attracted individuals already favorable to screening. Finally, survey translations from previous studies were not validated for comprehension in this population.

## 5 Conclusion

This study underscores the importance of developing communication strategies that are clear, accessible, and free of stigma. To be effective, these campaigns must rely on multiple channels and focus on the tangible benefits of early detection, while avoiding moralizing discourse.

Beyond communication, the success of the screening program will also depend on the active involvement of healthcare professionals, especially general practitioners, who are recognized as trusted figures in the decision-making process. Lastly, the sometimes-critical perception of eligibility criteria highlights the need for transparent pedagogy and a beneficiary-centered approach. By integrating these elements, the pilot program in the canton of Vaud could serve as a model for national implementation, built on trust, inclusivity, and responsiveness to the real needs of the target population.

## CRediT authorship contribution statement

**Mélinée Schindler:** Writing – original draft, Investigation, Formal analysis, Data curation. **Cynthia Schneider:** Writing – original draft, Formal analysis, Data curation. **Christophe von Garnier:** Writing – review & editing. **Cédric Bongard:** Writing – review & editing. **Jean-Luc Bulliard:** Writing – review & editing, Investigation. **Chiara Pozzessere:** Writing – review & editing. **Kevin Selby:** Writing – review & editing, Supervision, Methodology, Investigation, Conceptualization. **Christina Akre:** Writing – review & editing, Supervision, Methodology, Conceptualization.

## Funding

This work was supported by the Roberto and Gianna Gonella Foundation, Lausanne, Switzerland*.*

## Declaration of competing interest

The authors declare that they have no known competing financial interests or personal relationships that could have appeared to influence the work reported in this paper.

## Data Availability

Data will be made available on request.
